# Mental health help-seeking behaviour among migrant workers and migrant domestic workers in Singapore: a mixed-methods study

**DOI:** 10.1186/s13690-026-01844-z

**Published:** 2026-01-30

**Authors:** Mythily Subramaniam, Yunjue Zhang, Pratika Satghare, Yen Sin Koh, Anitha Jeyagurunathan, Yun Ting Lee, S Archana, M Iskandar Shah, Jason CH Yap, Chee Yong Lim, Lenny Azuree, Halina Talib, Siow Ann Chong

**Affiliations:** 1 Research Division, Institute of Mental Health, Buangkok Green Medical Park, 10 Buangkok View, Singapore, 539747 Singapore; 2 Saw Swee Hock School of Public Health, National University of Singapore, Singapore, 117549 Singapore; 3 Lee Kong Chian School of Medicine, Nanyang Technological University, Singapore, 308232 Singapore; 4 Foreign Manpower Management Division (FMMD), Ministry of Manpower, Singapore, 339946 Singapore; 5 Assurance, Care, Engagement (ACE) Group, Ministry of Manpower, Singapore, 339626 Singapore

**Keywords:** Asia, Barriers to care, Help-seeking behaviours, Mental health, Migrant workers

## Abstract

**Background:**

Migrant workers (MWs) and migrant domestic workers (MDWs) in Asia face elevated risks of mental health issues due to factors like long working hours, abuse, and family separation. In Singapore, despite established support systems, formal help-seeking remains low. This study aimed to investigate the mental health help-seeking behaviors, preferences, and perceived barriers and facilitators among MWs and MDWs in Singapore.

**Methods:**

A mixed-methods study was conducted in two phases. Phase 1 involved a quantitative survey with 1,465 MWs and 1,462 MDWs from the six largest nationality groups in Singapore. Phase 2 consisted of 14 focus group discussions (FGDs) with 48 MWs and 52 MDWs to explore their experiences in further detail.

**Results:**

The majority of MWs (79.2%) and MDWs (91.4%) reported seeking help for emotional problems, primarily from informal sources such as family and friends. Awareness of formal support services, such as the Ministry of Manpower (MOM), was moderate among MWs (58.4%) and MDWs (66.8%). For receiving mental health information, both groups preferred face-to-face workshops. However, their preferences for help-seeking differed; while 48.1% of MWs preferred a trained professional, 51.5% of MDWs preferred a trained fellow migrant worker. The qualitative findings identified significant barriers to formal help-seeking, including fear of job loss, financial constraints, lack of awareness of services, and stigma. Key facilitators included trustworthy relationships with family and friends, and positive, culturally sensitive interactions with healthcare professionals.

**Conclusions:**

While many MWs and MDWs experience emotional distress and seek support, they predominantly rely on informal networks due to multi-level barriers to formal care. These findings underscore the need for integrated support models that include peer-led initiatives, culturally and linguistically competent healthcare services, and clearer communication about available, affordable mental health resources to improve the well-being of this population.

**Supplementary Information:**

The online version contains supplementary material available at 10.1186/s13690-026-01844-z.

**Table:** 

Text box 1. Contributions to the literature
• There is limited research on barriers to mental health help-seeking among low-wage migrant workers in high-income Asian settings.
• Help-seeking preferences differ by migrant subgroup, with male workers favouring professionals and female domestic workers relying more on peers.
• Peer-led support models are viewed as acceptable and trusted pathways to improve access and reduce stigma in this population.
• Despite awareness of available services, fear of job loss remains a key structural barrier deterring formal help-seeking.

## Background

Between 1990 and 2020, Asia experienced a 93% increase in its intraregional migrant population, growing from 35.5 million to 68.5 million. The most notable growth in intraregional migration occurred in Southeast and West Asia, where migrant movements rose by 280% and 214%, respectively, over the same period [1]. Today, the Asia–Pacific region hosts around 24 million migrant workers, many of whom work in low-wage, labour-intensive, and precarious jobs [1]. While they contribute significantly to both origin and host economies [2], they face heightened risks of depression, anxiety, and trauma-related disorders driven by long hours, poor living conditions, abuse, and prolonged separation from family [3–5].

Singapore is home to approximately 456,800 migrant workers (MWs) in the construction, marine shipyard, and process sectors, and about 301,600 migrant domestic workers (MDWs), according to data as of December 2024 [6]. To safeguard their welfare, Singapore has established various regulatory frameworks and protective measures. These include mandatory orientation programmes for employers of migrant domestic workers (MDWs) and settling-in programmes for newly arrived non-Malaysian MWs. Additionally, the country has made notable strides in enhancing workplace health and health regulations. As of April 2022, employers are required to purchase a Primary Care Plan (PCP) as part of the work pass requirements for workers living in dormitories or working in the construction, marine shipyard, and process sectors. The PCP covers unlimited acute or chronic medical consultations and treatments, as well as 24/7 telemedicine services. MWs may also seek care at any designated general practitioner clinic in partnership. Furthermore, employers need to increase medical insurance coverage to $60K for their migrant employees. Several Voluntary Welfare Organisations (VWOs), such as HealthServe, also provide support to migrant workers through health education talks, helplines, individual counselling, group therapy, and crisis debriefs [7]. However, the effectiveness of these policies in addressing mental health needs remains understudied and largely unknown, particularly in terms of how intersectional factors like gender, nationality, and job type would shape their help-seeking behaviour.

Formal help-seeking from mental health professionals is low among MWs, where several intersecting barriers deter them from accessing care [4, 8–10]. These barriers operate at the individual (e.g., language barriers), interpersonal (e.g., fear of reputational damage), societal (e.g., stigma around mental illness), and structural levels (e.g., risk of employment termination). Understanding the interplay of these barriers is crucial for designing equitable, responsive mental health policies and outreach efforts. Studies suggest that informal social networks act as a source of emotional, informational, and instrumental support for MWs [11, 12]. Some turn to religious and spiritual practices, faith-based organisations, and embassies or consulate services when facing psychological distress [13] - settings that, although supportive, typically do not involve professional mental health care providers.

The current study aimed to understand mental health help-seeking behaviors among MWs and MDWs in Singapore using a mixed-methods approach. The study quantitatively explored workers’ awareness of organisations or agencies they could seek help from; the people, agencies, or organisations they had sought help from; and the ways they would like information on mental health conveyed to them. A deeper understanding of their perceived barriers and facilitators to accessing care, and the ways in which they had sought help for their emotional problems, was qualitatively examined through focus group discussions (FGDs). Given the timing of data collection during the early implementation phase of the PCP, this study provides a valuable baseline to inform future monitoring and evaluation efforts.

## Methods

### Study design

This mixed-methods study comprised two phases. In Phase 1, the MWs and MDWs from the six largest nationality groups in Singapore: Indian, Chinese, and Bangladeshi for MWs, and Filipino, Indonesian, and Burmese (citizens of Myanmar) for MDWs were surveyed to understand the extent of psychological distress, awareness of places they could seek help from and places that they had sought help from for their emotional and psychological problems. In Phase 2, the research team conducted a qualitative study to gain an in-depth understanding of the barriers and facilitators to help-seeking and how they had sought help for emotional and mental health problems.

### Study setting and participants

To meet the inclusion criteria, all MWs had to be within the legal working age of 18–60 years, while MDWs had to be within the age range of 23–60 years, set by the Ministry of Manpower, Singapore (MOM). MWs needed to work in the construction, marine shipyard, and process sectors and stay in dormitories during participation. MDWs who participated in the study had to be working in Singapore at the time of recruitment.

MWs were recruited through convenience sampling at designated dormitories and recreation centers (*n* = 1,465), while MDWs were surveyed at popular gathering locations on weekends (*n* = 1,462). The research team first conducted surveys with migrant workers (MWs) and subsequently contacted those who expressed interest in the qualitative component team to schedule focus group discussions (FGDs). Following the completion of survey data collection with MWs, the team undertook the same process for migrant domestic workers (MDWs). Data collection for MWs took place from August 2022 to July 2023, while data collection for MDWs was carried out between March and November 2023.

### Procedures

Experienced researchers who had worked on large-scale epidemiological and qualitative studies and were fluent in the participants’ native languages conducted the quantitative and qualitative interviews. In addition, the study team engaged and trained volunteers fluent in Bengali and Burmese to administer the questionnaire, ensuring that the workers’ language needs were fully addressed. Participants were informed that their responses would be anonymous and that participation would have no impact on their employment or access to services. To minimize concerns regarding confidentiality and access to identifiable data, verbal informed consent was obtained in place of written consent. All participants received a brochure with help-seeking information, and those who exhibited signs of distress were supported through established follow-up protocols, including immediate access to mental health services. The study received ethical approval from the National University of Singapore Institutional Review Board (NUS-IRB, Ref. No: NUS-IRB-2021-724).

#### Phase 1 quantitative survey

Socio-demographic data were collected through a structured questionnaire at the onset of the survey. The data included age, gender, ethnicity, marital status, education level, number of children, working arrangements, and employment history. Additionally, data on awareness of organisations where they could seek help for their problems while working in Singapore, whether they had sought help for their emotional issues, and if they had, where they had sought help, how they would like to receive information about mental health, and whom they would want to get help for their emotional problems were collected. The questionnaire was translated into the relevant native languages of the workers (i.e., Tamil, Mandarin, Bengali, Tagalog, Bahasa Indonesia, and Burmese). Thus, they could answer the questionnaires in English or their native language.

#### Phase 2 focus group discussions

After obtaining consent for the Phase 1 quantitative survey, the research team enquired about the willingness of MWs and MDWs to participate in the Phase 2 FGDs. They were informed that the FGDs aimed to delve deeper into the themes of experiences related to help-seeking for mental illness in Singapore. The study team randomly selected and contacted those who were willing to be recontacted for the FGDs. In all, 14 FGDs were conducted (7 per group, labelled in the order they were conducted), involving 48 MWs and 52 MDWs. The FGDs were conducted on a predetermined date and at a suitable venue (e.g., a dormitory, a multi-purpose hall at a recreational centre, etc.). FGDs for each nationality were conducted in their native languages, except for MDWs from the Philippines, who preferred to converse in English. For MDWs from Myanmar, FGDs were conducted in English and Tamil with those who could speak either language. Trained qualitative interviewers conducted the FGDs, which lasted approximately 1 to 1.5 h each. The FGDs continued until the team agreed that they had reached data saturation. In other words, when no new themes or new content in existing themes emerged from the FGDs.

### Analysis

#### Statistical analysis

The quantitative results were summarized using frequencies and percentages. All analyses were performed using SPSS Statistics software version 23.0 (IBM Corp, Armonk, NY, US) and Stata/MP version 18.0.

#### Qualitative semi-structured interview guide

An interview guide was designed to explore MWs’ and MDWs’ perceptions of mental health and their experiences in accessing healthcare in Singapore. It covered a range of topics, including their views on mental illness, words or phrases used to describe mental illness, and factors affecting help-seeking. For this study, we focused only on barriers and facilitators of help-seeking for mental health problems and sources of help.

#### Qualitative data analysis

Practical thematic analysis was adopted for reading and coding the data, following the approach of Saunders et al. [14]. All the FGDs were transcribed verbatim and checked for accuracy by a study team member. For non-English FGDs, transcripts were translated into English before analysis.

Seven members of the research team (MS, YZ, PS, AJ, YTL, SA, and MIS) with diverse disciplinary and cultural backgrounds reviewed the transcripts to familiarise themselves with the data. Two additional team members with relevant language expertise (FD and RT) coded the transcripts conducted in Bahasa Indonesia and Tamil, respectively. These language-specific transcripts were then translated and reviewed in discussion with the wider team to ensure fidelity and consistency in thematic interpretation. The researchers wrote memos (i.e., brief notes on thoughts and questions) and a summary of their impressions of the data, coded the transcripts assigned to them independently, and identified key themes. To ensure consistency and reliability, a codebook was developed listing code labels, comprehensive descriptions for each code, and examples observed in the data. This process was iterative, with the researchers meeting multiple times to finalise the codebook. After finalizing the codebook, researchers used it to assign codes to the data. They continued adding codes for any new concepts observed in the data. Throughout the coding process, the researchers met regularly to reconcile their differing knowledge and understandings of migrant workers’ culture and lived experiences. In addition, they highlighted new codes and discussed any text that they found ambiguous or difficult to code. Codes derived directly from the data were then grouped into meaningful themes, i.e., ‘meta-constructs that rise above codes and unite the dataset’ [14], reflecting recurring patterns across participant narratives. Although some resulting themes aligned with known topical domains (e.g., stigma, cost, lack of awareness), these were not pre-imposed categories but rather grounded in participants’ own framing of barriers. The coding was conducted manually.

## Results

### Quantitative survey

#### Socio-demographic characteristics and help-seeking behavior of migrant workers

Data from 1465 MWs were included in the analysis. Table 1 shows the socio-demographic characteristics of the MWs included in the study. Most of them were 30–39 years old (42.5%, *n* = 622), had secondary school educational qualification (47.8%, *n* = 700), had 7–14 years of education (78.4%, *n* = 1148), were married (72.7%, *n* = 1065), with children (66.1%, *n* = 969) and non-smokers (64.1%, *n* = 938).

**Table 1:** Socio-demographic characteristics of migrant workers

	*n*	%
Age Group		
20–29	390	26.6%
30–39	622	42.5%
40–49	352	24.0%
50–62	101	6.9%
Educational Qualification		
Primary and below	105	7.2%
Secondary	700	47.8%
Pre-Tertiary Education	504	34.4%
Tertiary Education	151	10.3%
Others	4	0.3%
Years of education		
≤ 6 years	79	5.4%
7–14 years	1148	78.4%
≥ 15 years	237	16.2%
Marital Status		
Married	1065	72.7%
Single	375	25.6%
Separated/Widowed/Divorced	20	1.4%
Co-habiting	5	0.3%
With Children		
Yes	969	66.1%
No	496	33.9%
Smoking status		
Non-smoker	938	64.1%
Smoker	374	25.5%
Former smoker	152	10.4%

Missing data: Educational Qualification (*n* = 1), Years of education (*n* = 1), Smoking status (*n* = 1)

About 58.4% (*n* = 855) of MWs were aware of organisations for help-seeking when they faced problems while working in Singapore. Among these participants, 76.1% (*n* = 651) mentioned MOM; 10.9% (*n* = 93) mentioned the police, and 5.15% (*n* = 44) mentioned their Embassy as organisations where they could seek help.

**Fig. 1:**
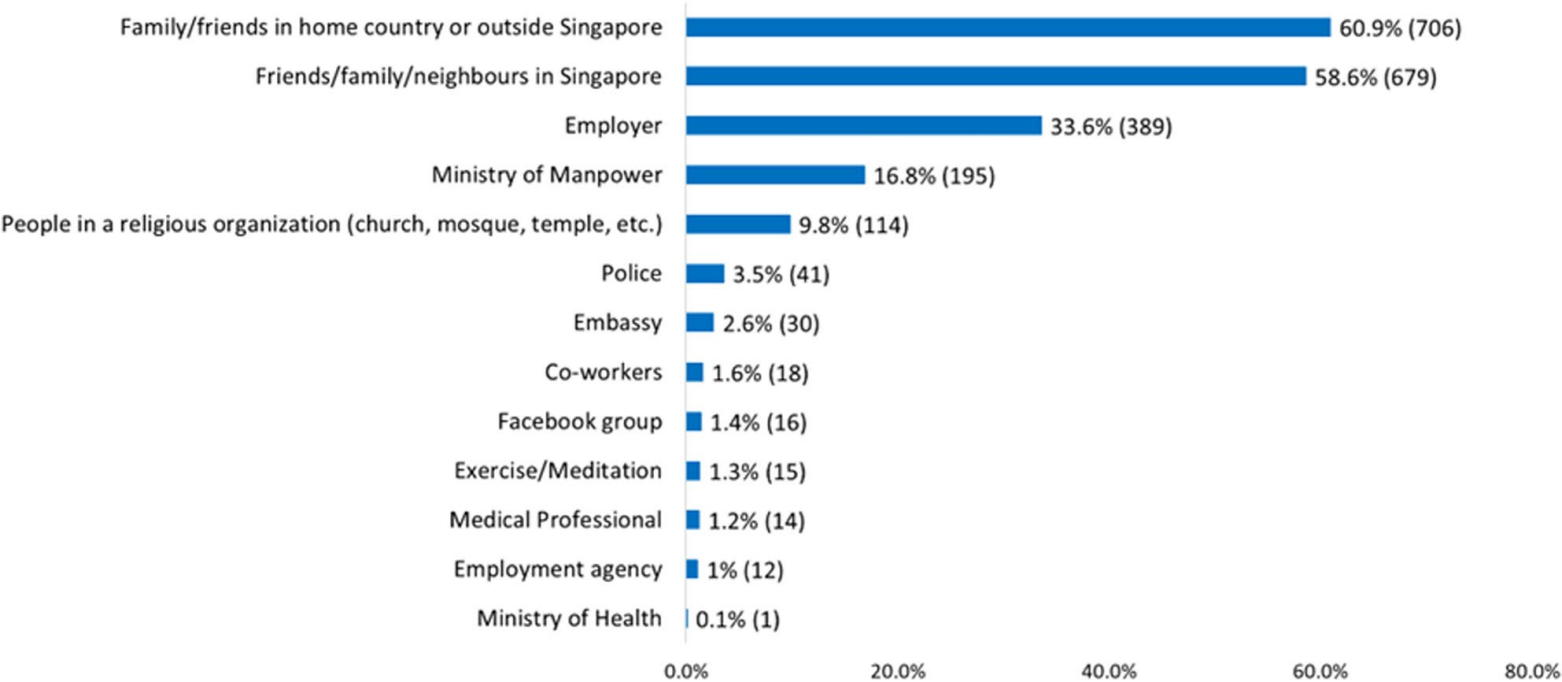
Sources of emotional support utilised by migrant workers

In all, 79.2% of MWs (*n* = 1159) had sought help from someone for their emotional problems; 60.9% (*n* = 706) had sought help from their family and friends who were in their home country or outside Singapore, while 58.6% (*n* = 679) reported seeking help from friends/families/ neighbours in Singapore, while 33.6% (*n* = 389) had sought help from their employer. Please see Fig. 1 for sources of support for emotional problems among MWs.

**Fig. 2:**
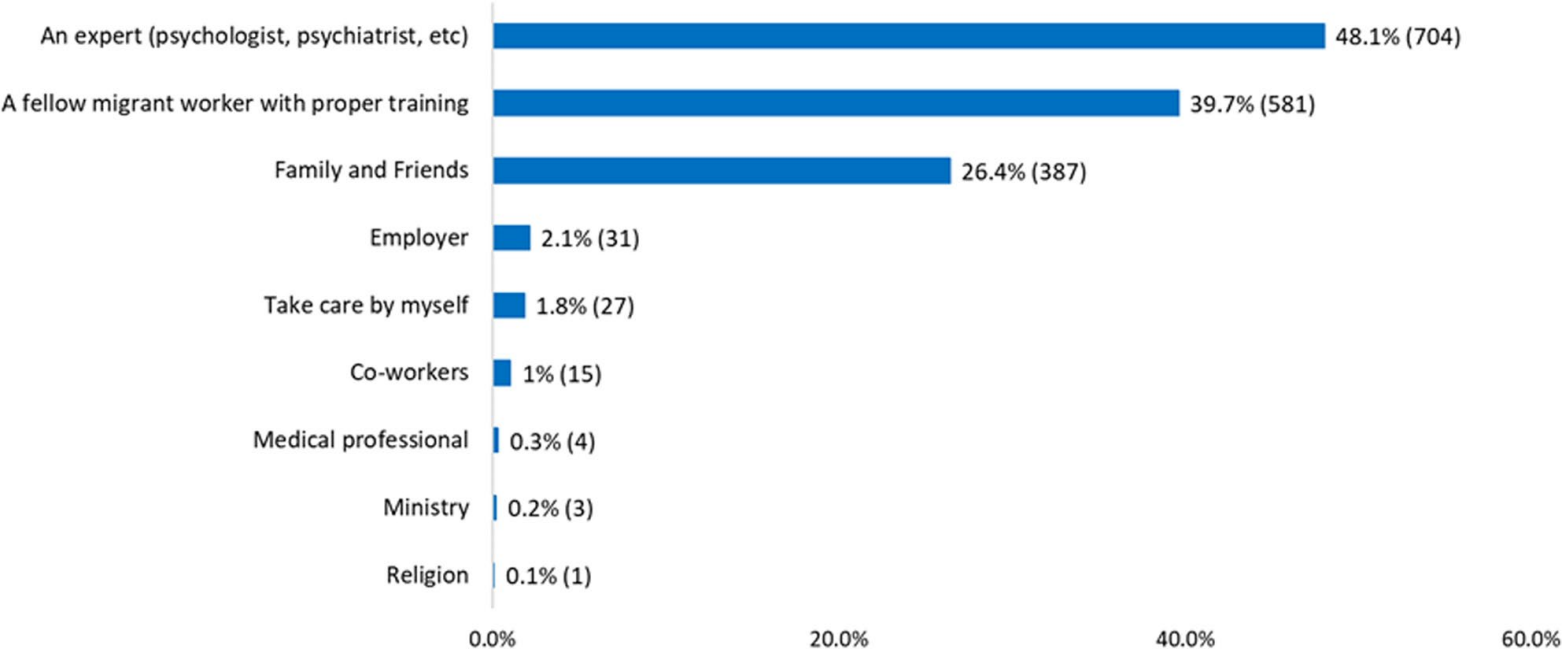
Help-seeking preferences for emotional problems among migrant workers

The majority of MWs (69.1%, *n* = 1012) stated that they would like to obtain information about emotional problems from face-to-face workshops or seminars, while 35.3% (*n* = 517) said that they would like such information from printed material such as leaflets, flyers, etc. (Supplementary Fig. 1a). When asked to identify whom they would like to seek help from for their emotional problems, 48.1% (*n* = 704) stated that they wanted to receive such assistance from a trained professional, like a psychiatrist or a psychologist, while 39.7% (*n* = 581) stated that they would like to receive such help from a trained fellow migrant worker. Please see Fig. 2 for preferred sources of help-seeking among MWs.

#### Socio-demographic characteristics and help-seeking behavior of migrant domestic workers

A total of 1462 MDWs completed the survey. Table 2 shows the socio-demographic characteristics of the MDWs included in the analysis. Most of them were aged 30–39 years old (39.5%, *n* = 577), had secondary school educational qualification (56.1%, *n* = 820), had 7–14 years of education (87.3%, *n* = 1262), were single (39.8%, *n* = 580), had children (66.3%, *n* = 966) and were non-smokers (97.9%, *n* = 1393).

**Table 2:** Socio-demographic characteristics of migrant domestic workers

	*n*	%
Age Group		
20–29	247	16.9%
30–39	577	39.5%
40–49	521	35.6%
50–62	117	8.0%
Educational Qualification		
Primary and below	85	5.8%
Secondary	820	56.1%
Pre-Tertiary Education	418	28.6%
Tertiary Education	136	9.3%
Others	3	0.2%
Years of education		
≤ 6 years	117	8.1%
7–14 years	1262	87.3%
≥ 15 years	67	4.6%
Marital Status		
Married	547	37.5%
Single	580	39.8%
Separated/Widowed/Divorced	327	22.4%
Co-habiting	5	0.3%
With Children		
Yes	966	66.3%
No	492	33.7%
Smoking status		
Non-smoker	1393	97.9%
Smoker	3	0.2%
Former smoker	27	1.9%

Missing data: Years of education (*n* = 16), marital status (*n* = 3), With children (*n* = 4), smoking status (*n* = 39)

In all, 66.8% (*n* = 977) of the MDWs were aware of organizations for help-seeking when they faced any problems while working in Singapore. Among these participants, 75.4% (*n* = 737) mentioned MOM, 46.8% (*n* = 457) mentioned the Centre for Domestic Employees (CDE), and 12.5% (*n* = 122) mentioned their employment agency as places where they could seek help.

**Fig. 3:**
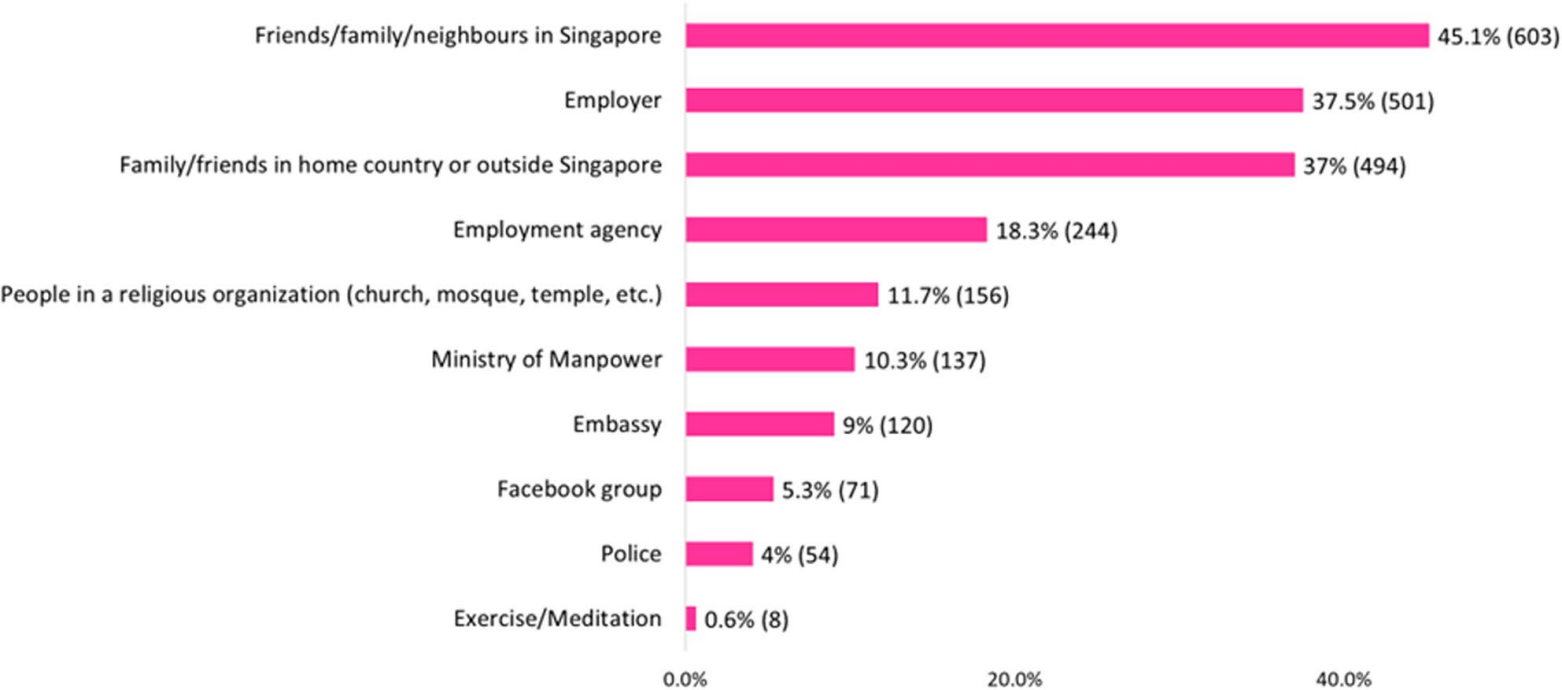
Sources of emotional support utilised by migrant domestic workers

The majority of MDWs (91.4%, *n* = 1336) got help when they had an emotional problem. Among those who had sought help, the top three sources of help were friends/family/neighbours in Singapore (45.1%, *n* = 603), employers (37.5%, *n* = 501), and friends in their home country or outside Singapore (37.0%, *n* = 494). Please see Fig. 3 for the sources of support for emotional problems among MDWs.

**Fig. 4:**
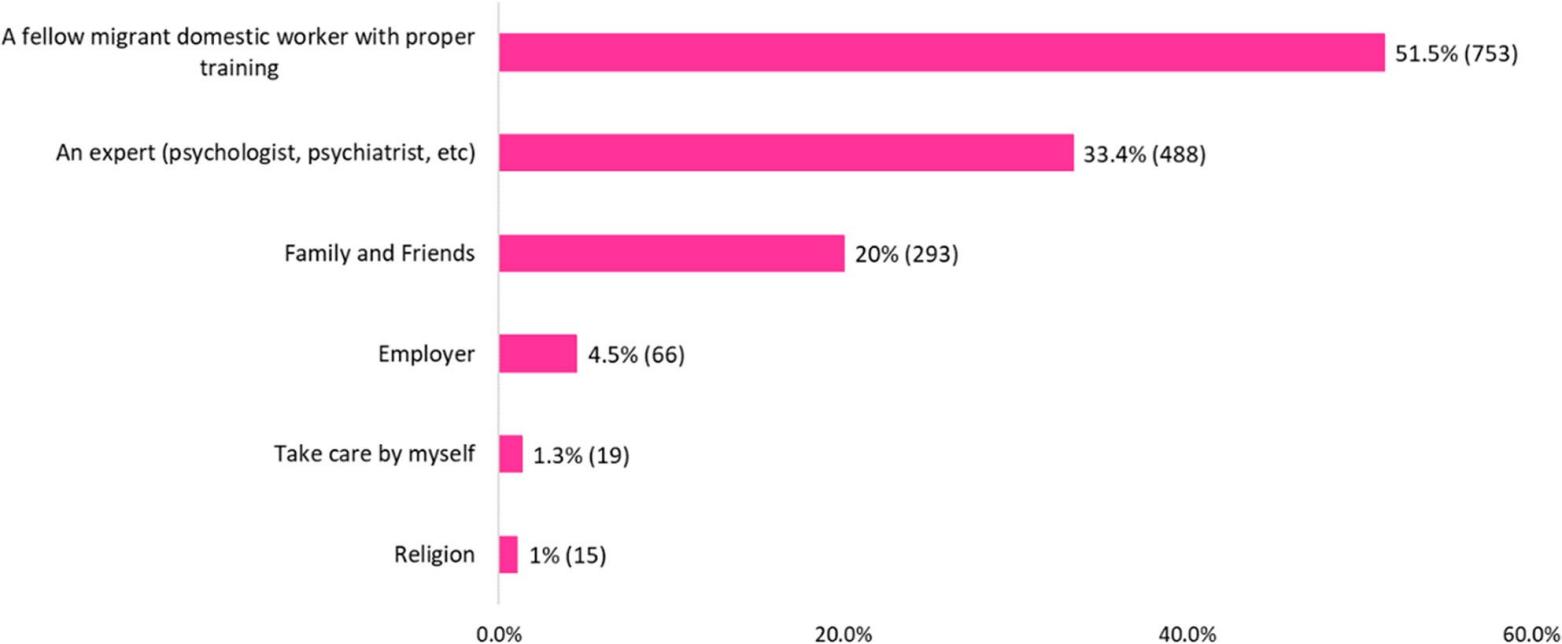
Help-seeking preferences for emotional problems among migrant domestic workers

About 81.0% (*n* = 1185) of the MDWs wanted information about their emotional problems from face-to-face workshops or seminars, while 18.9% (*n* = 277) wanted it through printed materials such as leaflets or flyers (Supplementary Fig. 1b). While 51.5% (*n* = 753) of MDWs wanted to talk to a fellow migrant domestic worker who was trained, 33.4% (*n* = 488) wished to speak to a trained professional (psychiatrist or psychologist) for their emotional problems. Please see Fig. 4 for preferred sources of help-seeking among MDWs.

### Qualitative – focus group discussion

As the themes and sub-themes that emerged from MWs and MDWs were broadly similar across the two groups, they are presented together. Three major themes were identified from the data collected from both MWs and MDWs: *Barriers to help-seeking for mental illnesses*, *Facilitators to help-seeking for mental illnesses*, and *Sources of help-seeking for mental illnesses* in Singapore.

## Barriers to help seeking for mental illness

A common theme among both MWs and MDWs was the multiple barriers they face in seeking help with mental health issues. These barriers included:

### Individual-level barriers

#### Lack of awareness of available services

Lack of awareness about existing mental health services emerged as a key individual-level barrier, as they were unfamiliar with the local healthcare systems. As one MW explained, *“They don’t know where to go. I think that could be one of the major reasons.”(MWs FGD 4)* While some MWs knew who they should approach for seeking mental health problems, in their home countries, they struggled to identify comparable resources locally. As stated by another MW, when asked about sources of help-seeking, *“Doctor only should say. One who deals with mental health…No*,* I was talking about in my native place. I don’t know anyone here.” (MWs FGD 2)*.

This distinction illustrates how specific knowledge of service pathways in one’s home country does not translate to the host country, resulting in a gap in help-seeking even when distress is recognised. As a result, some relied on their embassies as a safety net. One MDW highlighted this gap, noting that while the Embassy is a known entity, it remained an untested resource for mental health for her: *“I think the main reason that is we don’t know. We don’t know who to go to*,* we don’t know who to approach to*,* and we don’t know anything. I know we say the Philippine Embassy. I haven’t been to a Philippine embassy to seek help*,* but as far as I know*,* there’s a lot of people there seeking help.” (MDWs FGD 5).* This suggests that while awareness of general support structures (such as embassies) is high, it does not necessarily translate into accessible or specialized mental healthcare pathways. However, we acknowledge that, while workers often internalised this lack of awareness as a personal limitation, it also reflects broader systemic gaps in providing the necessary information, especially in participants’ native languages.

#### Communication difficulties

Beyond language, workers cited difficulties initiating conversations about personal issues, particularly with unfamiliar individuals. As one worker noted: *“We can’t just go to someone (who is not known to us before) and ask or talk. In those situations*,* we may feel like outsiders.” (MWs FGD 2)* The perceived status of being an “outsider” created psychological distance and distrust, making formal services, which are often transactional, feel inaccessible or intimidating, regardless of language fluency.

#### Expectations of self-reliance

Some workers were encouraged to resolve personal issues independently. They noted that while they were advised to turn to supervisors for work problems and family for personal ones, there were no clear pathways for mental health concerns, further reinforcing an internalized sense of self-reliance. This led many to self-manage their distress rather than seeking external support. As one MDW described:

*“I can manage stress like now*,* my experience*,* this is now mine too. Uh*,* sometimes*,* uh*,* with even cooking*,* singing*,* sometimes even in front of the two grandma and grandpa. I am dancing*,* dancing with them*,* laughing with them*,* then they laugh also. All de-stress and this stress is gone. [laughs] That’s-that’s why*,* No. Because I don’t want to share my– I don’t want to share*,* uh*,* even to my friends. I didn’t want to share my problem. I can solve by my own.*”( *MDWs FGD 6*).

This narrative reveals a strong sense of resilience, which was equated with solving the problem internally. By successfully using distraction techniques (singing and dancing), the MDW reinforced the idea that external help was unnecessary. The explicit refusal to share suggests that self-reliance serves as a protective mechanism, potentially preventing them from seeking professional help; however, participants were unwilling to discuss the alternatives once self-management strategies failed.

### Interpersonal-level barriers

#### Fear of gossip and social judgment

Concerns about gossip and isolation reinforced their reluctance. Many felt that help-seeking efforts could isolate them from their only established social networks within their country of work (i.e., with their employer and fellow domestic worker friends). As a result, many expressed that they were unwilling to take the risk.

*“Others would do something to us*,* like that. They will laugh.” (MWs FGD 2)*.

*“As one of my relatives said*,* we value our reputation too much.” (MWs FGD 6)*.

The explicit mention of “reputation” suggested that for many migrant workers, social capital within their community was an important resource. Consequently, the risk of losing this standing through gossip outweighed the potential benefits of seeking professional help.

### Societal-level barriers

#### Stigma

Many participants highlighted the fear of being stigmatized as a significant deterrent when considering seeking support or treatment for mental health issues. Specifically, many were worried that others might misjudge them for seeking professional mental healthcare services. This stigma was experienced as a tangible fear that the label of “madness” would lead to immediate social exclusion. Consequently, they preferred to preserve their social standing by remaining silent rather than leveraging their personal support networks to overcome their mental health concerns.

*“Some things can be shared with others*,* while others cannot*,* especially with relatives. They will name us also as mad…He is mad. We shouldn’t talk too much with them.” (MWs FGD 2)*

Moreover, participants feared that this stigma would impact their professional lives, where the label of mental illness carries the weight of incompetence. In particular, MDWs were worried that their employer would view them through a lens of prejudice rather than empathy, prioritizing stigma over their mental health needs.

*“There is also*,* um– ’cause me*,* I’m scared also that my employer will*,* like– it’s….Yeah*,* because mental…. crazy).” (MDWs FGD 1)*

*“You go to see to their mental health doctor or something*,* so they will think is you are crazy already.” (MDWs FGD 3)*

### Structural/ institutional- level barriers

#### Fear of loss of work opportunities or termination

Fear of job loss was a dominant concern. Many participants expressed anxiety that disclosing mental health struggles might lead to contract termination, non-renewal of work permits, or future exclusion from employment in Singapore. As one MW explained, the fear of administrative repercussions often outweighed the need for care:

*“There is a mental health place*,* but why don’t they go there? They have fear*,* whether they would submit it to the authorities*,* that they won’t get their renewal*,* this is the only reason and main reason.” (MWs FGD 3)*

This reflects the precarious nature of migrant employment, where health status is often equated with work eligibility. Many participants perceived help-seeking not as a medical decision, but as a risk that could alert authorities and jeopardize their legal right to remain in the country.

*“Here working in Singapore*,* you don’t know if employer is going to kick you out when you tell your problem to them.” (MDWs FGD 5)*

Participants viewed the time required for therapeutic interventions as fundamentally at odds with the rigid demands of their work schedule. Help-seeking was thus feared as a trigger for immediate repatriation, as it was seen as invalidating the worker’s purpose in their employer’s assessment.

*“To the doctor for counselling*,* it will take a week. We may face workplace issues like that. Not getting a salary or else being laid off by the company too.” (MWs FGD 2)*

*“She will*,* uh*,* uh– she will say*,* “Oh*,* you’re crazy already. You go back to Philippines.” Like that.” (MDWs FGD 1)*

#### Financial barriers

Both MWs and MDWs cited the cost of mental healthcare as a significant deterrent. In the absence of employer support, subsidies, or comprehensive insurance, professional treatment was perceived as financially inaccessible. This barrier was further amplified by the workers’ significant remittance obligations to families in their home countries. Consequently, participants often viewed healthcare expenditure as a trade-off against financial survival, deliberately prioritising savings over seeking treatment. These deep-seated concerns regarding the affordability of care were echoed across multiple participants.

*“To save money*,* because in our perception*,* no matter who we turn to for help*,* it’s going to cost some money*,* especially the hospitals. Many people are trying to save money*,* and they’d think a lot.” (MWs FGD 4)*

*“Last time I don’t have money*,* I never go see a doctor. I ask my friend*,* also don’t have money*,* he never see a doctor.” (MWs FGD 5)*

*“Money also*,* you need money….(to seek treatment)” (MDWs FGD 5)*

*“Me*,* I’m scared about expenses*,* or. Because this isn’t…. it’s not covered in our insurance*,* right*,* mental illness?” (MDWs FGD 5)*

#### Healthcare system-related barriers

Participants reported discomfort discussing mental health issues with healthcare providers, citing a lack of inquiry about mental well-being and perceived indifference. They also appeared to value a holistic approach familiar to them from home, whereas they perceived the system in Singapore as more focused on physical care, which discouraged them from raising non-physical complaints.

*“They haven’t asked all those things.” (MWs FGD 2)*

*“Not here. Most of the doctors in our town will ask about our mental health. In our native place.” (MWs FGD 2)*

Language barriers also exacerbated the problem, as many workers had limited English proficiency and feared being misunderstood. This inability to communicate created a power imbalance and a feeling of helplessness, discouraging workers from attempting to navigate the formal health system.

*“Yes. What happened*,* I can tell them. In Singapore*,* I know a little bit of English only and then I cannot tell properly*,* and doctor also don’t understand me.” (MDWs FDG 3)*

#### Lack of institutional support (MDWs)

This sub-theme emerged exclusively among MDWs. Participants expressed a lack of support from their embassies and local VWOs during their help-seeking journey. They shared specific challenges they experienced, such as their mental health concerns being dismissed as attention-seeking behaviour and receiving critical and disparaging responses that blamed them for their struggles. Such negative encounters with gatekeepers are particularly damaging, as they invalidate the workers’ concerns and fears and discourage future help-seeking. The following statements exemplify the lack of support.

*“You are just acting because you just want attention. No*,* they will just think*,* oh*,* are acting up.”(MDWs FGD 5)*

*“You did something wrong*,* that’s why you come here for help (expressed by embassy staff to MDWs seeking help from them). Something like that.” (MDWs FGD 5)*

## Facilitators for help-seeking for mental illness

Despite the presence of barriers hindering access to and utilisation of formal mental health services, participants discussed key enablers that allowed them to obtain help. These comprised trustworthy relationships, positive experiences with healthcare professionals, exercise or meditation, social support, and personal strengths.

### Trustworthy relationships (family/friends)

Some participants expressed that they would be more willing to ask friends or family for help with whom they had close, trusted relationships. This reliance on “kith and kin” underscores that trust is a prerequisite for disclosure.

*“If you are my friend or relative*,* I can ask for help…” (MWs FGD 2)*

Additionally, social engagement itself served as an active coping strategy. By externalizing their worries through conversation, participants shared how they transformed personal problems into a shared burden, which facilitated emotional relief.

*“Sometimes I go out … to forget the thing that– to talk to different people. Share also with your friends*,* what you have*,* that’s good. Then the friends also can help you like that.” (MDWs FGD 5)*

### Positive experiences with healthcare professionals

MWs felt that regular contact with familiar doctors, especially those who spoke their native language, enhanced a sense of safety and made them more comfortable sharing mental health concerns, suggesting that linguistic congruence facilitated more than communication and created a culturally safe environment.

*“He is a Tamil Doctor. When we go there*,* he would ask what our problem is*,* whether it is a fever*,* a cold*,* or something else*,* we are gonna tell him our problem.” (MWs FGD 2)*

Some doctors proactively asked about mental health issues, which also helped workers feel more comfortable disclosing, highlighting the importance of provider-initiated inquiry. Because workers often lacked the confidence or vocabulary to raise these issues themselves, their doctor’s proactive questioning legitimized mental health as a valid medical concern.

*“We will tell them if they asked. Some doctors will ask whether we are having some mental issues.” (MWs FGD 2)*

## Sources of help-seeking

The MWs and MDWs identified several informal and formal sources that supported their efforts to seek help for mental health challenges. These included informal sources such as interpersonal relationships (family and friends) and religious practices, as well as formal sources such as mental health services available in Singapore.

### Family and friends

Speaking with family members and friends, both in Singapore and in their home countries, was a crucial form of emotional support. Participants reported that simply talking about their problems with friends, children, or family members back home would ease their minds and relieve stress. Verbalizing their distress acted as a form of catharsis. Informal networks provided workers with a safe space for emotional release, helping prevent the accumulation of psychological stress.

*“So*,* to ensure my mental health*,* I talk to my kids.” (MWs FGD 2)*

*“Yes*,* I will share with my friends*,* after sharing my mind will be free.” (MWs FGD 3)*

### Religious and spiritual support

MWs and MDWs stated that they found comfort and healing through religious practices and visiting places of worship. Faith functioned not just as a ritual, but through surrendering control to a higher power; workers found the psychological strength to endure hardships that were otherwise beyond their control.

*“We can go to the Temple.” (MWs FGD 3)*

*“More important is*,* just pray. Trust God. All this*,* yeah. That is my…. that’s– I go through. Yeah.” (MDWs FGD 1)*

*“Talking in a good way*,* uh*,* praying to God*,* and*,* uh*,* nothing is impossible if you pray…” (MDWs FGD 6)*

Other narratives illustrated a coping style centered on acceptance and resilience. Religious belief provided a broader perspective, helping workers normalize their struggles and encouraging them to persevere.

*“Just move on. We are not the only one. All people are suffering*,* so we must move on and pray. Then whatever problems*,* let go and move on.” (MDWs FGD 6)*

### Migrant worker support and advocacy networks in Singapore

Some participants were aware of available formal resources and able to name them. Some carried cards with printed information on where to seek help for stress and emotional problems. Specific agencies mentioned by them included the Migrant Workers’ Centre (MWC) and the Assurance, Care and Engagement (ACE) Group under the Ministry of Manpower. One worker shared that the MWC’s helpline enabled those who were in mental distress to share their problems and receive the support and help they needed. MDWs also mentioned CDE and Aidha as organizations that offered comprehensive support for help-seeking, including counselling, guidance, and informative workshops on essential skills such as stress management and financial literacy.

*“MWC contact number because of the mental issue or feeling that he is alone; they have their contact number. In the call*,* he can talk to them*,* he can share with the MWC people.” (FGD 5 MWs)*

*“CDE…Yeah. Like*,* they’re offering like guidance*,* how can you -- overcome your thoughts*,* your whatever. Maybe then can help you to– advise you*,* like.” (MDWs FGD 1)*

Online organisations on social media served as valuable resources for MDWs looking for support. As these communities do not have access limitations, domestic workers could easily receive peer support and obtain other helpful information, such as details on hotlines and local support groups.

*“Also*,* on Facebook*,* there are organizations such as*,* uh*,* Facebook for PMI (Indonesian Migrant Workers) for TKW (Female Workers) to share anything.” (MDWs FGD 2)*

A couple of participants mentioned specialist facilities, such as psychological services available in Singapore, where one can seek help for any mental health complaint.

*“There are a lot of places in Singapore…psychiatrists*,* almost in every big public hospital in Singapore …. You can go for counselling*,* for minor problems. There are lots of them….”* (*MWs FGD 4*)

### Employers

This theme was largely unique to MDWs. Many MDWs emphasized the importance of openly expressing mental health concerns to their employer. Due to daily interactions, employers were seen as readily accessible outlets for emotional expression, especially in times of urgent need, when families and friends may not be physically present to provide necessary care. The theme underscores the unique live-in nature of domestic work. Unlike other sectors, the domestic worker’s workplace is also their home, making the employer the most immediate and sometimes only source of tangible support during a crisis, recognizing that the employer acts as the primary safety net in the host country.

*“Maybe that’s the best way. You need to talk to your boss*,* or if you feel something like that*,* talk to them or call your family. “I feel something like this*,* if you’re working here in Singapore*,* if something happens*,* you need to talk to your employer.”(MDWs FGD 5)*

### Self-care and self-reliance

Some MWs shared that they turned to personal strategies, such as exercise or meditation, to cope with stress and encouraged their peers to do the same. These behavioral strategies served a dual purpose; they were physically restorative and socially connective. By inviting peers to “walk” or “talk,” workers broke the cycle of isolation, replacing it with low-barrier, peer-led coping mechanisms.

*“I’ll even ask*,* “How is it going? Let’s go for a walk*,* or let’s talk. I will try to give him company.”(MWs FGD 2)*

*“They should do meditation.”(MWs FGD 2)*

Others expressed confidence in their ability to manage emotional challenges independently, emphasizing personal resilience and emotional control. This narrative of self-reliance served as a protective cognitive strategy. By framing stress as a manageable aspect of their life rather than a medical illness, workers preserved their sense of agency. Normalizing the distress allowed them to maintain their primary identity as providers without adopting the potentially stigmatizing role of a mentally ill person.

*“No Sir*,* I will manage. I have self-esteem and trust*,* so I don’t have stress and hard feelings.” (MWs FGD 2)*

*“We are all here for earning*,* we all left family and native and all*,* everybody has that feeling*,* but we are not exposing that! When we feel stressed*,* we will talk over the phone…These feelings and all not an illness*,* we will manage to do it.”(MWs FGD 2)*

## Discussion

Our study provides important insights into help-seeking behaviors and the perceived barriers and facilitators to mental health support among MWs and MDWs in Singapore. Both the quantitative and qualitative findings reveal that while a majority of MWs and MDWs are aware of both formal and informal avenues for help, they tend to seek help from informal sources, and multiple individual, societal, and systemic barriers continue to hinder formal help-seeking for mental health concerns. These findings offer an early snapshot of mental health literacy, help-seeking preferences, and perceived barriers in the immediate aftermath of the PCP rollout and can serve as a baseline reference for future research and policy evaluation.

### Help-seeking behavior and preferences

A notable proportion of both MWs and MDWs reported seeking help for emotional problems - predominantly from informal sources such as friends, family, and employers. This pattern mirrors previous findings suggesting that migrant workers often prefer informal over formal mental health support due to trust, cultural familiarity, and accessibility [13, 15]. Formal support systems, characterized by institutional regulations and standard operating procedures, are typically task-oriented and leverage professional expertise to address mental health issues. This structure ensures consistent and standardized service delivery; however, it may sometimes lack the flexibility to cater to individual needs, potentially leading to perceptions of impersonal care, and may seem inaccessible, transactional, and uncaring to MWs and MDWs [16]. In contrast, informal support sourced from family, friends, and community members is often more accessible and emotionally attuned, providing spontaneous, personalized assistance that resonates with the individual’s unique circumstances [17]. While informal support plays a crucial role in offering emotional comfort and a sense of belonging, it may not always provide the appropriate structured interventions necessary for managing severe mental health conditions.

Notably, the strong preference for receiving mental health information through face-to-face workshops rather than written materials underscores the importance of culturally sensitive, interactive modes of outreach for this population. The preference for face-to-face sessions may also highlight the linguistic and socio-cultural barriers that limit migrant workers’ ability to access and navigate online mental health resources independently. It would be valuable to pilot visual-based interventions to enhance their knowledge and awareness of mental health conditions and available services in Singapore. Research indicates that visual interventions are effective mainly because they promote greater interactivity and minimize reading demands [18, 19]. Delivering these materials in the workers’ native languages could further improve their accessibility and impact.

While nearly half of MWs and MDWs expressed a preference for seeking help from trained professionals, a considerable proportion were also open to receiving support from trained fellow migrant workers. A previous study in Singapore demonstrated that peer support interventions were effective in improving the mental health of migrant construction workers [20]. These findings suggest that it is feasible to expand peer-support models to support both MWs and MDWs. However, it is crucial to provide adequate training and ongoing support for peer supporters to prevent potential distress and burnout [11].

### Barriers to help-seeking

Our qualitative findings highlight that barriers to mental health help-seeking among MWs and MDWs are multi-layered. Key concerns included the fear of job loss, financial constraints, gaps in mental health literacy, both in terms of lack of awareness of services and stigma and barriers related to healthcare.

Fear of termination or contract non-renewal if mental health issues were disclosed emerged as a dominant concern. This is consistent with the existing literature, which shows that employment insecurity is a significant deterrent to health disclosure among migrant populations [21]. The precariousness of migrant work contracts, often tied to employer sponsorship, and the pressure workers feel to maintain current and future employment to support their families amplifies these fears and discourages timely intervention.

Many workers were unaware of available mental health services in Singapore or believed such services would be prohibitively expensive. Importantly, misconceptions regarding healthcare coverage for mental health issues suggest a critical need for more transparent communication. Mental illness stigma, both self-perceived and anticipated from others, strongly influenced reluctance to seek help. Participants expressed the fear of being labeled “mad”, being gossiped about, or socially ostracized. These findings parallel broader local and international evidence that stigma within migrant communities remains a powerful barrier to mental healthcare access [18, 22, 23].

Communication difficulties and culturally incongruent healthcare experiences further inhibited help-seeking. Migrant workers frequently report lower satisfaction with healthcare encounters in host countries due to linguistic barriers and perceived provider indifference [23, 24]. Participants in our study emphasized a preference for providers who could speak their native language and who would proactively address mental health.

### Facilitators to help-seeking

Despite the barriers, several facilitators to mental health help-seeking were also identified. Trustworthy relationships with family and friends were key enablers, consistent with social support theories highlighting the protective role of close interpersonal networks [17]. Positive experiences with healthcare professionals, particularly when language and cultural familiarity were present, also encouraged help-seeking behaviors. Religious and spiritual practices provided emotional resilience for many participants, a finding consistent with previous studies showing that faith-based coping mechanisms are integral to migrant well-being [25]. Finally, it was heartening to note that many workers felt that their employer could be a source of help or had sought help from their employer for their mental health problems.

The study has several policy and practice implications. These range from improving the mental health literacy of the migrant community by communicating with them through videos or face-to-face sessions on mental illnesses, the importance of early detection and treatment, and links to self-care resources. Training peer supporters from within migrant communities could bridge trust gaps and offer culturally safe pathways to care. Given the large number of migrant workers in Singapore, it is essential to build healthcare providers’ capacity to offer linguistically and culturally sensitive care to them. Finally, embassies and Voluntary Welfare Organizations should receive more resources and training to better support migrant mental health needs, as they remain a significant source of help-seeking.

Since 2020, Singapore has implemented structured programmes to support MWs’ mental well-being from arrival onward. Newly arrived MWs working in construction, marine shipyard, or process sectors attend the mandatory Settling-In Programme at the Onboard centre, which includes components on managing stress and adapting to working and living in Singapore. The programme further provides information about available support channels and resources they can access during their stay, including primary healthcare services. At the national level, Project DAWN (Depression Awareness, Well-being and Normalisation) was established as a framework to guide the development of a comprehensive mental health support ecosystem for MWs. This initiative focuses on raising mental health awareness among MWs and stakeholders, reducing stigma, and improving access to care services.

Several initiatives have also been launched to raise awareness of mental well-being among MDWs and their employers. All first-time MDWs must attend an MDW Settling-in Programme, where they are educated on stress management and effective communication. First-time employers are required to complete the Employer’s Orientation Programme to understand their responsibilities of care towards their MDWs. There are also “Care Sisters,” trained MDWs who empower and support fellow MDWs with their mental well-being or other challenges. MOM operates a helpline dedicated to MDWs in distress, which is publicised on social media to raise awareness.

### Limitations

This study had some limitations. Data was self-reported, which may introduce social desirability bias, especially around sensitive issues like mental health. Furthermore, the cross-sectional nature of the survey limits causal interpretations. Most of the FGDs were conducted in English, Tamil, and Bengali, and we may have missed information unique to other ethnic groups who were not comfortable speaking English.

The strengths of the study lie in the large sample size, the translation and administration of questionnaires in migrant workers’ local languages, and the assurance of confidentiality by not linking any identifiers to the collected data, which enabled the collection of rich and valid data. Furthermore, the use of a sequential mixed-methods design, culturally sensitive data collection protocols, and the triangulation of survey data with rich qualitative narratives strengthen the validity of the findings.

## Conclusions

Supporting and caring for the migrant workforce (MWs and MDWs) requires a whole-of-society effort. Partnerships with non-governmental organisations, healthcare providers, and employers are essential to ensure the programmes and services are coordinated and sustainable. Efforts to strengthen the mental health ecosystems of this population should also take into account the socio-cultural needs and unique challenges to ensure sustainability and effectiveness. 

## Supplementary Information

Supplementary Material 1: Migrant Workers’ Preferences for Accessing Information for Emotional Problems.

Supplementary Material 2: Migrant Domestic Workers’ Preferences for Accessing Information for Emotional Problems.

## Data Availability

The datasets generated and/or analysed during the current study are not publicly available due to ethical requirements and participants’ confidentiality concerns, but are available from the corresponding author upon reasonable request.

## Abbreviations

ACE: Assurance, Care and Engagement
CDE: Centre for Domestic Employees
FGDs: Focus Group Discussions
MDWs: Migrant domestic workers
MOM: Ministry of Manpower
MWC: Migrant Worker’s Centre
MWs: Migrant workers
PCP: Primary Care Plan
VWOs: Voluntary Welfare Organisations
