# Supplementary figures and images for: Mental health help-seeking behaviour among migrant workers and migrant domestic workers in Singapore: a mixed-methods study
Source: Arch Public Health. 2026 Jan 30;84:45. doi: 10.1186/s13690-026-01844-z (PMC12934044; doi:10.1186/s13690-026-01844-z)

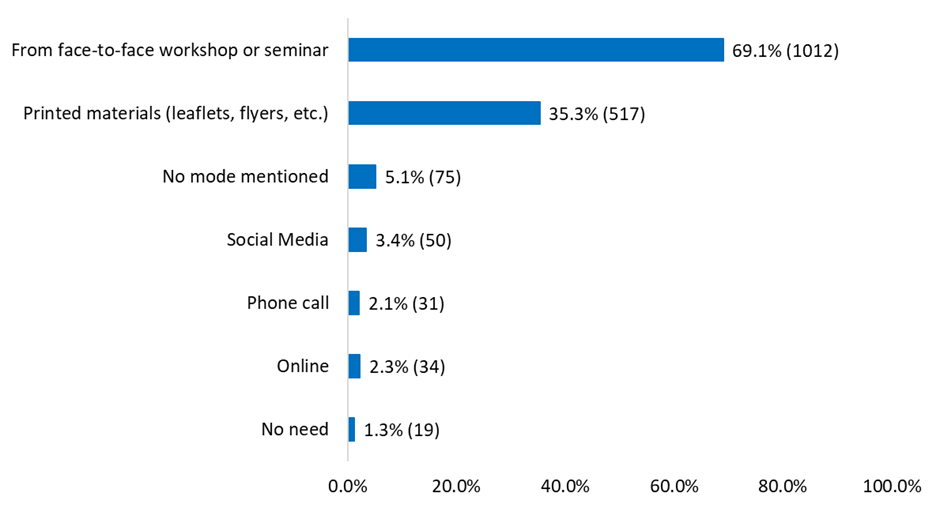

Supplement: Supplementary file 1 — Supplementary Material 1: Migrant Workers’ Preferences for Accessing Information for Emotional Problems. [file 13690_2026_1844_MOESM1_ESM.tif]

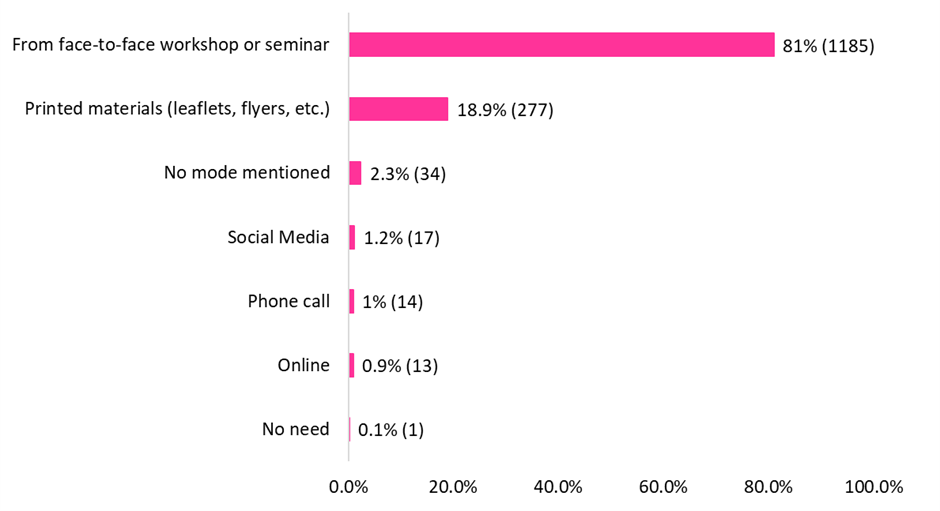

Supplement: Supplementary file 2 — Supplementary Material 2: Migrant Domestic Workers’ Preferences for Accessing Information for Emotional Problems. [file 13690_2026_1844_MOESM2_ESM.tif]
